# Review of Hypofractionated Radiotherapy for Prostate Cancer

**DOI:** 10.5402/2012/410892

**Published:** 2012-08-13

**Authors:** Despina Spyropoulou, Dimitrios Kardamakis

**Affiliations:** Department of Radiation Oncology and Stereotactic Radiotherapy, Medical School, University of Patras, 26504 Patras, Greece

## Abstract

Hypofractionated radiotherapy for prostate cancer has become of increasing interest with the recognition of a potential improvement in therapeutic outcome with treatments delivered in large-sized daily fractions. In addition, hypofractionation offers a reduction in fraction number and produces attractive cost and increased convenience for patients. There is convincing evidence, by several clinical trials, that biochemical control is significantly improved with higher administered radiation doses to the prostate gland. Furthermore, the improved radiation delivery techniques such as 3D conformal radiotherapy (3DCRT) or, better, intensity modulated radiation therapy (IMRT) allow high administered doses to the prostate while sparing the normal surrounding tissues. Several studies of the radiobiology of prostate cancer suggest that it may be more susceptible to large fraction sizes compared with conventional fractionation of external beam radiation.

## 1. Introduction

Besides the healthcare systems, prostate cancer is considered to have an impact on society as well, as far as biologic, economic, and personal parameters are concerned. External beam radiation therapy (EBRT) is one of the standard treatment modalities for treating patients with prostate cancer, and is often delivered conformally in order to spare as much the amount of radiation received by the surrounding normal tissues. It is estimated that about 30% of all prostate cancer patients will receive radiotherapy [1], and a significant number of these patients will eventually be cured.

Quality of life has become one of the most significant issues in treatment decisions in prostate cancer. While conventional fractionation radiation regimens are usually employing fractions of 1.8–2.0 Gy daily, hypofractionation, as the name suggests, refers to the delivery of radiotherapy dose in smaller number of treatments that would be used to deliver a traditional dosing scheme. The daily fraction size, therefore, is larger than the size given in standard fractionation. Hypofractionated external beam radiotherapy has been used clinically for a number of years, particularly in the UK [2–4]. Given that quality of life has become one of the most significant issues in treatment decisions in prostate cancer, hypofractionation offers a shorter treatment course and increases convenience for prostate cancer patients.

In radiobiology, the *α*/*β* ratio, defined as the dose at which killing of cell by linear (*α*) and quadratic (*β*) components are equal, is used to quantify the fractionation sensitivity of tissues and tumors. A low *α*/*β* ratio is consistent with a greater capacity for repair between fractions, with an accompanying greater relative sparing with small fraction sizes, than for tumors with their typically higher *α*/*β* ratios. Recent publications suggest that the *α*/*β* ratio of prostate cancer was comparable to that for late-responding normal tissue or even lower because of the slow natural turnover rates in a high proportion of these tumors [5]. Values of *α*/*β* ranging from 1.2 to 4 have been reported, while most data support values at the lower end of this spectrum [6–13]. An important implication of the low *α*/*β* of prostate cancer is that a favorable therapeutic outcome can potentially be achieved by delivering a smaller number of larger fractions. More specifically, if *α*/*β* ratio for prostate cancer is lower than the nearby normal tissues, then the therapeutic advantage can be gained by using fewer and larger fractions.

This paper examines the clinical experience with hypofractionated radiotherapy for prostate cancer. Published clinical data will be classified into four categories. The first category includes retrospective clinical trials that have been analyzed post hoc to evaluate *α*/*β* ratio for prostate cancer. The second category includes clinical trials of hypofractionated regimens that did not depend on the *α*/*β* ratio hypothesis. The third category includes prospective phase I to II studies using fractionation regimens that assumed that the *α*/*β* ratio for prostate cancer is low and the fourth category includes completed randomized clinical trials comparing different fractionation schedules.

## 2. Post Hoc Analysis of Retrospective Clinical Trials

The article concerned with the low *α*/*β* ratio hypothesis of prostate cancer was first published in 1999 by Brenner and Hall [14]. These authors analyzed 2 datasets of prostate cancer patients treated with radiation therapy to estimate the *α*/*β* ratio. One was using external beam radiation therapy and the other was using permanent brachytherapy. The authors used the standard linear-quadratic model and disease-free estimates relied on PSA values. The authors reported an estimated *α*/*β* ratio of 1.5 Gy. They concluded that the calculated *α*/*β* ratio argued for hypofractionation regimens using high-dose rate (HDR) brachytherapy or external beam radiation therapy. However, it was noticed that the clinical data used in this analysis presented three important weaknesses that must be considered. First, the datasets used in the modeling exercise were immature (potentially resulting in an overestimate of treatment efficacy that was influencing the results of the analysis). Second, the analysis relied on a short-term endpoint, and finally, the number of patients included in the analysis limited the precision of the disease-free estimates that were added to the model.

Attempting to phase these weaknesses, Brenner et al. [7] performed another post hoc analysis of a prospective dose escalation trial that combined external beam radiotherapy and HDR brachytherapy in men treated at a single institution. The authors used different regimens of high dose rate (HDR) given in either 2 or 3 implants to various doses. However, the authors relied again on a short-term endpoint (biochemical control at 3 years). From the original population of 192 patients, the authors managed to analyze the results in 121 men. They compared the 3-year biochemical control rates between a 2-implant group and a 3-implant group. Based on the observed difference between the 2 groups, the investigators derived an estimate of 1.2 Gy (95% confidence interval, 0.03–4.1) for *α*/*β*. The results of the clinical trial strongly supported evidence that *α*/*β* for prostate was “atypically” low. 

Brenner and Hall [14] suggested in 1999 that prostate tumors might not respond to changes in fractionation in the same way as other cancers. He hypothesized that prostate tumors might respond to changes in fractionation more like a late responding normal tissue. As though their suggestion was that the *α*/*β* ratio for prostate cancer might be low (1.5) comparable to that of late sequelae.

Recent analysis of clinical data by Fowler et al. [9], Brenner et al. [7], Bentzen and Ritter [6] showed remarkable agreement with the conclusion of Brenner and Hall's 1999 conclusions. These estimates are consistent with the very slow proliferation characteristics of prostate tumors in comparison with other malignancies. Most prostate tumors have an extremely low proportion of cycling cells with an average potential doubling time before treatment of 40 days ranging from 15 to more than 60 days, compared with about 5 days for many other types of tumors [5, 15, 16].

The peculiarity that the *α*/*β* ratio of prostate cancer is low implies that, theoretically, a hypofractionated radiation treatment schedule would increase the therapeutic gain of radiotherapy. Until now, following published randomized clinical trials, hypofractionated radiotherapy has shown results in terms of acute and chronic toxicity and tumor control similar to those obtained with conventionally fractionated radiotherapy.

## 3. Retrospective Clinical Trials with Hypofractionated Radiotherapy for Prostate Cancer

Hypofractionated radiotherapy regimens have been used for several decades, primarily due to the low availability of treatment machines. In 1991, Collins [17]reported on 232 patients treated over 20 years at Saint Thoma's Hospital in London. Patients enrolled in the clinical trial over a period of 1964 to 1984. The trial included men with early (T1-T2) and advanced (T3-T4) disease that were treated by external beam radiotherapy. Depending on anatomy, patients were treated with 3-field, 4-field, or a double rotation technique from a cobalt-60 machine or linear accelerator over three weeks with six 6-Gy fractions (2 fractions per week) for a total dose of 36 Gy. The therapy was generally well tolerated, and few patients had late complications. More specifically, two patients developed rectal strictures, and a few patients had recurrent rectal bleeding episodes. The conclusions stated that the hypofractionated radiotherapy regimen lead to comparable results to other reported series, whether assessed as a local response or by survival curves. 

Another series from the United Kingdom has been reported by Livsey et al. [18] at the Christie Hospital. The series included more than 700 men treated with hypofractionated radiotherapy over a period from 1995 to 1998 using 3.13 Gy fractions to 50 Gy. In this series, patients were stratified into low-, intermediate-, and high- risk disease based on clinical stage, PSA value, and Gleason score. Low-risk patients had clinical stage of T1 or T2, PSA less than or equal to 10, and Gleason score less than 7. If one criteria was exceeded, the patient was classified as intermediate risk; if two were exceeded, the patient was classified as high risk. Freedom from biochemical recurrence (FFBR) was calculated according to the American Society for Therapeutic Radiology and Oncology consensus definition [19]. Freedom from biochemical recurrence at 5 years was 82%, 56%, and 39% for the low-, intermediate-, and high-risk groups, respectively.

In order to estimate toxicity, patients were assessed by Radiation Therapy Oncology Group criteria for late urinary and rectal toxicity. Out of 101 evaluable patients, 9% experienced late grade 2 urinary toxicity, and 1% experienced late grade 3 urinary toxicity. The authors reported no grade 3 rectal toxicity. They concluded that the results were comparable to contemporaneous reports using more conventional fractionation regimens.

It is though important to mention that although these two clinical trials show low rates of morbidity, taking into consideration the long natural history of prostate cancer and the heterogeneity of patients within risk groupings, it is difficult to draw inference on treatment efficacy. 

## 4. Prospective Nonrandomized Clinical Trials of Hypofractionated Radiotherapy for Prostate Cancer 

Kupelian et al. [20] at the Cleveland Clinic were among the first that combined improvements in radiotherapy technology with the low *α*/*β* ratio hypothesis in order to examine a hypofractionated regimen in a controlled phase I/II clinical trial. The first conclusions on toxicity were reported in 2001, and the experience has been updated recently [21]. 

The authors used image-guided IMRT to treat men with localized prostate cancer using 2.5 Gy fractions to 70 Gy. The observed gastrointestinal and genitourinary morbidity in this trial was acceptable. The acute grade 2 urinary toxicity rate was 18%, whereas the acute grade 2 rectal toxicity was 9%. There was 1% grade 3 acute urinary toxicity, but no grade 3 acute rectal toxicity. At 5 years, the combined grade 2 to 4 late rectal toxicity was 4.5%. The authors calculated freedom from biochemical recurrence (FFBR) according to the ASTRO definition and the alternate nadir + 2 ng/mL definition. According to risk group, the FFBR estimates are 95%, 85%, and 68% for low-, intermediate-, and high risk patients, respectively using the ASTRO definition, and 94%, 83%, and 72%, respectively using the nadir + 2 definition. These results compare favourably to those achieved in patients contemporaneously treated with conventional fractionation at the Cleveland Clinic (78 Gy at 2 Gy/fraction).

Another study performed in 2005 by Tsuji et al. [22] reported on the National Institute of Radiological Sciences experience on 201 patients using carbon ion therapy in the hypofractionated treatment of prostate cancer during three clinical trials in Japan from June 1995 to February 2004. All involved patients had T1–T3 disease and were radiographically N0. After an initial dose-finding period, patients received daily fractions of 3.3 Gy equivalent, 4 days per week to a total dose of 66 Gy equivalent. The authors reported an overall 5-year FFBR rate of 83% with late grade 2 genitourinary and gastrointestinal toxicities of 6% and 1%, respectively. No grade 3 or higher toxicities were observed. The authors concluded that the regimen was effective and safe.

Furthermore, Soete et al. [23] reported on the results of a phase I/II study performed at 3 institutions in Belgium and Italy. The study included 36 patients treated with 56 Gy in 16 fractions (3.5 Gy per fraction) over 4 weeks, 4 fractions per week, using 3-dimentional conformal radiation therapy or IMRT. Patients were treated between April 2004 and May 2005. The objective of the trial primary endpoint was a comparison preliminary report focused on early toxicity using the RTOG/European Organization for Research and Treatment of Cancer (EORTC) acute toxicity grading system. No grade 3 or 4 acute toxicity was reported. The incidence of acute grade 2 gastrointestinal and genitourinary toxicity was 36% and 44%, respectively. The authors compared these toxicity results with a historical control group treated with conventional radiotherapy fractionation and found that the hypofractionation group experienced greater grade 1 to 2 toxicity (*P* < .01). The authors concluded that hypofractionation is feasible but that it is associated with higher rates of acute toxicity compared with patients treated with conventional fractionation.

Ritter et al. [24] reported a preliminary analysis of toxicity with hypofractionated radiotherapy at the 2006 ASTRO meeting. This collaborative effort reported on 110 patients treated with image-guided IMRT to a total dose of 64.68 Gy in 2.94 Gy fractions given 4 to 5 days per week. With a median followup of 20 months, the estimate of grade 2 rectal bleeding was 12% with no grade 3 rectal toxicity while no late genitourinary (GU) toxicity was reported.

## 5. Prospective Randomized Clinical Trials Comparing Different Radiation Regimens

Two randomized, controlled trials that have been published compared radiation regimens with different daily fraction sizes in patients with clinically localized prostate cancer. One trial took place in Canada and the other in Australia.

More specifically, Lukka et al. [25] reported on the results of PR 05, a Canadian randomized trial of 936 patients treated with either 66 Gy in 2 Gy fractions (long arm) or 52.5 Gy in 2.625-Gy fractions (short arm). The clinical trial was performed from 1995 until 1998 and patients were enrolled at sixteen centres in Canada. At 5 years, the biochemical/clinical failure rate was higher in the hypofractionated arm compared with the conventionally fractionated arm (hazard ratio = 1.18; 95% confidence interval, 0.99–1.41). However, there was no difference in overall survival between the two arms. Acute genitourinary toxicity was higher in the hypofractionated arm with 9% versus 5% grade 3 or higher. The observed acute gastrointestinal toxicity was also higher in the hypofractionated arm at 4% versus 3% grade 3 or higher. There were no statistically significant differences in late toxicity between the 2 arms. The incidence of late grade 3 or higher genitourinary or gastrointestinal toxicity was 1% and 2%, respectively.

The second, smaller, and randomized clinical trial was performed by Yeoh et al. [26]. The authors of this trial compared a conventionally fractionated regimen (2 Gy/fraction to 64 Gy) with a hypofractionated regimen (2.75 Gy/fraction to 55 Gy). The primary endpoint of the trial was to compare the late gastrointestinal and genitourinary toxicity. The trial was performed over a period of 7 years from 1996 to 2003 at a single centre. Patients were required to have TI or T2 disease, but there were no PSA or Gleason score requirements. Results from 217 patients were analyzed; 109 men received the conventional radiotherapy regimen, and 108 were treated with the hypofractionated radiotherapy regimen. The authors observed differences in toxicity depending on the treatment regimen. More specifically, acute rectal toxicity was worse in the hypofractionated arm, while late urinary and rectal toxicity were reported as equivalent but were not quantified. The freedom from biochemical recurrence was no different between the two arms (56% at 5 years). Furthermore, they also reported an equivalent overall survival rate of 85% in both hypofractionated and conventional regimen.

Furthermore, another prospective phase III randomized clinical trial by Arcangeli et al. [27], compared hypofractionation versus conventional fractionation in patients with high-risk prostate cancer. The purpose of this study was to compare the toxicity and efficacy of hypofractionated (62 Gy/20 fractions/5 weeks, 4 fractions per week) versus conventional fractionation radiotherapy (80 Gy/40 fractions/8 weeks). From January 2003 to December 2007, 168 patients were randomized to receive either hypofractionated or conventional fractionated schedules of three-dimensional conformal radiotherapy to the prostate and seminal vesicles. Considering the results of the trial, no difference was found for late toxicity between the two treatment groups, with 3-year grade II rates of 17% and 16% for gastrointestinal and 14% and 11% for genitourinary in the hypofractionation and conventional fractionation groups, respectively. However, the 3-year freedom from biochemical failure rates were 87% and 79% in the hypofractionation and conventional fractionation groups, respectively (*P* = .035).

## 6. Conclusions 

In recent years there has been increasing interest in hypofractionated radiotherapy for prostate cancer. A significant number of reports have suggested that *α*/*β* ratio for prostate cancer is low. If *α*/*β* ratio for prostate cancer is lower than *α*/*β* of the surrounding normal tissues (bladder and rectum), then hypofractionated regimens should result in an improved therapeutic outcome. There is now convincing evidence that biochemical control is improved with higher cumulative radiation doses to the prostate. In addition to the possible radiobiological benefits, hypofractionated radiotherapy with fewer fractions allows for increased patient convenience and minimal disruptions to their lives. Results from several clinical studies of hypofractionated radiotherapy, especially with IMRT, have found that late gastrointestinal and genitourinary toxicity is well tolerated. Another potential benefit of hypofractionated radiotherapy is reduction in treatment cost and shortening of waiting lists in high-volume radiotherapy departments.
